# Ecological conditions determine extinction risk in co-evolving bacteria-phage populations

**DOI:** 10.1186/s12862-016-0808-8

**Published:** 2016-10-24

**Authors:** Rosanna C. T. Wright, Michael A. Brockhurst, Ellie Harrison

**Affiliations:** Department of Biology, University of York, York, YO10 5DD UK

**Keywords:** Antagonistic coevolution, Experimental evolution, *Pseudomonas fluorescens*, Bacteriophage, Extinction, Species interaction

## Abstract

**Background:**

Antagonistic coevolution between bacteria and their viral parasites, phage, drives continual evolution of resistance and infectivity traits through recurrent cycles of adaptation and counter-adaptation. Both partners are vulnerable to extinction through failure of adaptation. Environmental conditions may impose unequal abiotic selection pressures on each partner, destabilising the coevolutionary relationship and increasing the extinction risk of one partner. In this study we explore how the degree of population mixing and resource supply affect coevolution-induced extinction risk by coevolving replicate populations of *Pseudomonas fluorescens* SBW25 with its associated lytic phage SBW25Ф2 under four treatment regimens incorporating low and high resource availability with mixed or static growth conditions.

**Results:**

We observed an increased risk of phage extinction under population mixing, and in low resource conditions. High levels of evolved bacterial resistance promoted phage extinction at low resources under both mixed and static conditions, whereas phage populations could survive when phage susceptible bacterial genotypes rose to high frequency.

**Conclusions:**

These findings demonstrate that phage extinction risk is influenced by multiple abiotic conditions, which together act to destabilise the bacteria-phage coevolutionary relationship. The risk of coevolution-induced extinction is therefore dependent on the ecological context.

## Background

Microbial communities are subject to a broad range of biotic and abiotic selection pressures and are capable of rapid evolutionary change in response [1]. Antagonistic interactions, such as between bacteria and bacteriophages (phages), in particular drive continuous evolutionary change through recurrent cycles of adaptation and counter-adaptation, known as Red Queen coevolution [2–4]. This process may be driven by directional selection favouring ever broader ranges of resistance and infectivity achieved by recurrent selective sweeps (Arms Race dynamics (ARD)) [5], or proceed by sustained allele frequency oscillations driven by negative frequency-dependent selection (Fluctuating Selection dynamics (FSD)) [6]. The stability of coevolutionary interactions relies upon interaction symmetry, both in terms of the selection acting upon each partner and correspondingly their potential for adaptation [4]. Failure of either partner to adapt quickly enough could result in their extinction [7]. Host-parasite communities are vulnerable to host extinction through failure to evolve resistance against increased parasite virulence [8], whereas parasite extinction could result from either an inability to overcome evolved host resistance [9], or via extinction of the host. The risk of extinction may be dependent on the pattern of coevolutionary dynamics, which have been shown to vary depending on ecological conditions [10, 11]. Strong directional selection under ARD coevolution may exacerbate asymmetries in evolutionary potential between the coevolving partners (e.g. phage infectivity evolution requires more mutational steps than reciprocal bacterial resistance evolution [12]) driving the more constrained partner to extinction. We are interested here in how ecological context, and associated abiotic selection pressures, influences the risk of coevolution-induced extinction.

Although not extensively investigated, recent studies suggest that abiotic environmental conditions can affect coevolution-induced extinction risk [9, 13, 14]. Phage adapting to deteriorating thermal conditions, for example, were able to adapt to thermal stress when their bacterial host was held in evolutionary stasis, but were driven to extinction in the presence of co-evolving hosts [13]. Additional selection from gradually increasing temperatures, to which phage are far more sensitive, constrained the phage’s ability to adapt to increased bacterial resistance, leading to extinction of the phage population [13]. Imbalanced antagonistic coevolution can therefore increase the extinction risk for phage populations, however it is unclear how this varies with the intensity of coevolution. Numerous ecological factors are known to alter the strength of reciprocal selection driving bacteria-phage coevolution. Increasing resource supply, by supporting larger population sizes, increases bacteria-phage encounter rates [15], and has been shown to drive phage extinction in both liquid media [9] and in soil [14]. However, the effects of nutrient enrichment go beyond demography; increased resource availability reduces the fitness costs associated with host resistance [9]. There is a documented trade-off between bacterial resistance and growth rate [16], which is fundamental to the maintenance of diversity in microbial communities, by preventing highly competitive and resistant host strains from dominating the population [17]. The allocation of this trade-off varies across environmental conditions; when resource availability is high more energy can be allocated to resistance mechanisms [18], effectively reducing the fitness costs associated with bacterial resistance. Therefore, it is challenging to distinguish the impact of intensity of coevolution from the effect of reduced fitness costs in driving phage extinction in nutrient enriched conditions.

Population mixing [19] accelerates coevolution in a similar way to increased resource availability [15]; increased bacteria-phage encounter rates strengthen reciprocal selection for the evolution of increased resistance and infectivity in bacteria and phage populations respectively. However, whereas increased resource availability mitigates the fitness costs associated with bacterial resistance [9, 15], population mixing does not. In contrast, unmixed populations experience reduced encounter rates due to the development of spatial and temporal host refuges against phage infection [20].

In this study, we explore how two ecological variables altering the intensity of bacteria phage coevolution, population mixing and resource level, affect the extinction risk of coevolving bacteria-phage populations in the *Pseudomonas fluorescens* SBW25 – lytic phage SBW25ɸ2 model system [21]. We predicted that environments supporting more intense coevolution, i.e. well-mixed, high resource supply environments, would be those most likely to drive coevolution-induced extinction. To test this hypothesis we experimentally coevolved replicate bacteria-phage populations by serial transfer under high or low resource supply, with or without population mixing in a full factorial experimental design and monitored population densities of each species over time.

## Methods

### Culture techniques

All cultures were grown in microcosms (30 ml glass universal bottles with loose fitting plastic lids) containing 6 ml of either high or low nutrient level media. Low nutrient level was obtained by 100-fold dilution of standard (high resource level) King’s Media B (KB) into M9 salt solution. Cultures were grown at 28 °C in either a static incubator or orbital shaker set to 200 rpm for 1 min in every 30 min (mixed treatment). Cultures were propagated by serial transfer, sub-culturing 1 % into fresh media every 48 h, for twenty transfers.

### Experimental design

Four treatments were established consisting of high and low resource levels with mixed and static environments of each. Independent clones of *P. fluorescens* SBW25 and bacteriophage SBW25φ2 were isolated and used to found 24 replicate populations (4 treatments × 6 replicates). Each replicate microcosm was inoculated with 10^8^ *P. fluorescens* SBW25 cells and 10^6^ SBW25φ2 particles. Bacteria and phage densities were calculated every second transfer; bacterial density as colony forming units (CFU/ml) by plating diluted cultures on KB agar plates, and phage density as plaque forming units (PFU/ml) by plating a serial dilution of filtered culture on a soft agar lawn of ancestral bacteria.

### Sampling techniques

At every second transfer whole population samples, phage populations and 20 individual bacterial clones were isolated and stored at -80 °C in glycerol solution (20 %). Phage populations were obtained by filtration (0.22 μm filters) to remove bacteria.

### Resistance and infectivity profiles approaching phage extinction

To investigate the mechanism of phage extinction in low resource level treatments, we characterised bacterial resistance and phage infectivity levels within each population approaching the phage extinction time point. The transfer at which early phage extinction events were most common was designated as the ‘extinction time point’ (*T* = 4 for low static; *T* = 6 for low mixed), and the previous sampling point as the ‘pre-extinction time point’ (*T* = 2 for low static; *T* = 4 for low mixed). Twenty phage clones per replicate were isolated from phage population glycerol stocks of the pre-extinction time point by plating phage filtrate on 0.8 % agar containing exponentially growing ancestral bacteria. The twenty bacterial clones from each low resource level replicate at the pre-extinction time point (henceforth ‘contemporary bacteria’) and at the extinction time point (henceforth ‘future bacteria’) were each assayed for resistance against their respective pre-extinction phage set.

Assays were performed in 96 well microtitre plates containing standard concentration of KB media in M9 salts for all treatments. Phage were added at a density approximately 10 times higher than bacterial density (multiplicity of infection, MOI = 10). The absorbance at 600 nm was measured at *t* = 0 and *t* = 20 h to give a relative bacterial growth rate (RBG) [22] compared to a control in the absence of phage, given by Eq. 1.

For phage *i*, bacteria *j*:

1 $$ {\mathrm{RBG}}_{ij}=\frac{{\left[ Ab{s}_{600}\left(t=20\right)- Ab{s}_{600}\left(t=0\right)\right]}_{ij}}{{\left[ Ab{s}_{600}\left(t=20\right)- Ab{s}_{600}\left(t=0\right)\right]}_{controlj}} $$

The threshold for binary infection outcome was calculated by modelling a normal distribution over the resistance peak of the RBG distribution and taking the 95 % confidence interval (RBG = 0.781). The proportion of possible pairwise interactions within each replicate population (20 bacteria clones × 20 phage clones = 400 possible interactions) that resulted in infection (RBG < 0.781), describes qualitative population-level susceptibility. This is referred to (e.g. Fig. 3) as the ‘Proportion of realised interactions’. Link strength (1-RBG) of realised interactions measures quantitative susceptibility at the individual level, such that 0 describes completely resistant bacteria and 1 completely susceptible bacteria.

### Statistical analysis

Phage extinction was analysed using a survival model (fitted to a parametric Weibull distribution) on the time of extinction taken as the time point phage became undetectable in each replicate, with censoring to account for phage populations which survived the entire experiment. Analysis of the bacterial densities over time was performed using a generalised least squares (GLS) linear model with auto-correlation over time within replicates on log_10_ transformed CFU values. In this model, time, presence or absence of phage, nutrient level and mixing status were given as fixed effects. Individual linear mixed effect models were made for both population-level (proportion of realised interactions) and individual-level (link strength) susceptibility as the response variable, fitting mixing, phage extinction status and bacterial time point (contemporary or future) as interacting fixed effects, and replicate population as a random effect. Tukey post hoc testing was used to isolate the drivers of significant main effects.

## Results

**Fig. 1:**
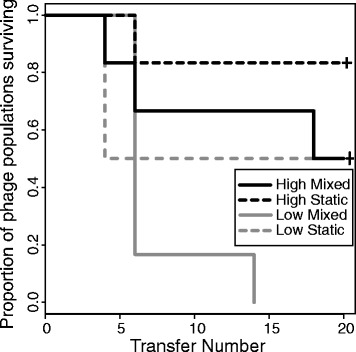
Survivorship of phage replicate populations by treatment. Survivorship is given as the proportion of replicate populations within each treatment in which phage are present at each transfer. Phage populations were judged to be extinct if phage were undetectable by plating undiluted filtrate of the respective replicate population

**Fig. 2:**
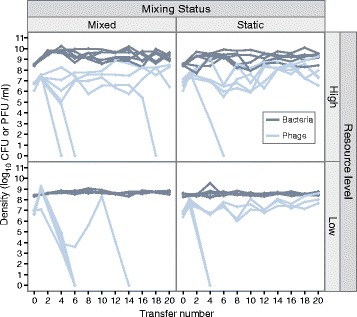
Population densities of bacteria and phage within replicate populations over time. Phage densities (light *blue*) denote plaque forming units (PFU/ml) and bacterial densities (*dark blue*) are colony forming units (CFU/ml). Different lines represent the 6 replicate populations within each treatment

Out of six replicate populations per treatment, phage extinction was observed in all low resource replicates under population mixing and half of those in static conditions. Phage extinction was less common in high resource conditions, with only one extinction event in static conditions, and three out of six replicates in mixed populations (Fig. 1). The likelihood of phage extinction was increased by low resource levels and population mixing (survival model: resource level *Z* = -2.59, *p* = 0.00954; population mixing *Z* = -2.28, *p* = 0.0226). There was no significant interaction between these two treatments (survival model: interaction between resource and mixing *Z* = 0.106, *p* = 0.915); the individual effects were additive to give the highest likelihood of phage extinction in the low resource, mixed environment. In contrast, no bacterial population extinctions were observed. Bacterial population densities were approximately 4-fold higher in high compared to low resource environments whilst population mixing had a small effect, increasing bacterial density by approximately 45 % (GLS: high resource *t* = 9.83, *p* < 0.0001; mixing *t* = 2.36, *p* = 0.0185; Fig. 2).

**Fig. 3:**
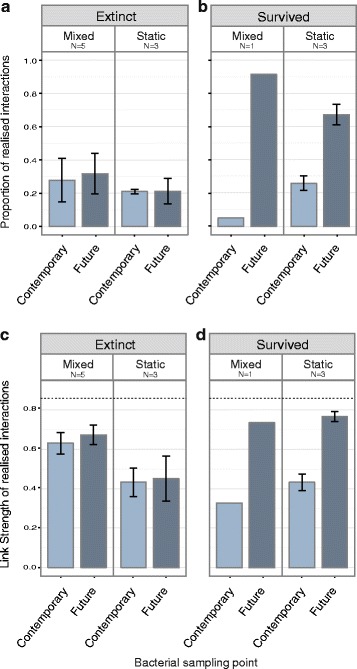
Bacterial susceptibility across low resource treatments approaching phage extinction time points. **a, b** mean (± SE) proportion of realised interactions (using binary threshold of susceptibility) and (**c, d**) mean (± SE) link strength of realised interactions within replicate populations of mixed and static low resource treatments. Bacteria sampled at pre-extinction time points (Contemporary) or extinction time points (Future) assayed against phage sampled from the same replicate population at the pre-extinction time point. Designated ‘Extinct’ (**a, c**) or ‘Survived’ (**b, d**) based on phage presence at transfer 10. ‘N’ describes the number of replicate populations represented under each heading. The dashed line in **c**, **d** shows the link strength of ancestral phage against ancestral bacterial

Extinction events occurring in low resource populations were further investigated by estimating the susceptibility of bacterial populations to infection by phage clones from their sympatric populations approaching the extinction time points. Bacterial susceptibility was characterised by two distinct measurements. Firstly, population-level susceptibility is described by the proportion of realised interactions on a scale from 0, where all bacteria sampled are resistant to infection from all phage sampled, to 1, indicating all bacteria were susceptible to all phage (i.e. ancestral level susceptibility). The second metric, link strength, gives an estimate of the mean quantitative susceptibility of bacteria, measured as the reduction in bacterial growth caused by phage infection, for each replicate population. We observed high levels of resistance to phage from the pre-extinction time point in contemporary (pre-extinction) bacteria irrespective of whether phage subsequently survived or became extinct (Fig. 3a, b). There was no difference in the proportion of realised interactions between bacterial sampling points for extinction populations (Fig. 3a; PRI Tukey *p* = 0.9036). However, in replicates where phage survived beyond the time-point where extinctions were observed in other replicates, bacteria sampled from the extinction time point (future) were significantly more susceptible to infection by pre-extinction time point phage (Fig. 3b; PRI Tukey *p* < 0.0001). In addition, increased link strength in future compared to contemporary bacteria in survival populations indicated greater quantitative susceptibility at the individual level (Fig. 3d; LS Tukey *p* = 0.0337) suggesting that phage survival relied upon the reinvasion of highly phage-susceptible bacterial genotypes. This pattern was consistent for both mixed and unmixed environments. Population mixing had no significant effect on proportion of realised interactions in extinction populations (Fig. 3a, PRI Tukey *p* = 0.9551). However, link strength was greater in mixed than static conditions (Fig. 3c, LS Tukey *p* = 0.0410), indicating population mixing promoted higher individual-level susceptibility in extinction populations.

## Discussion

We have found that low resource conditions and population mixing both act to increase the likelihood of phage extinction. These findings, in part, contradict our predictions that more intense coevolution, known to be accelerated by both high resource availability and high rates of population mixing, should promote coevolution-induced phage extinction.

Our predictions were based on previous studies that found elevated phage extinction in nutrient-enriched environments using the same bacteria-phage model system [14], which Buckling and Zhang formalised as a ‘coevolutionary paradox of enrichment’ [12]. They hypothesised that increasing nutrient levels can destabilise the coevolutionary interaction between bacteria and phage by increasing the intensity of antagonistic coevolution, selecting for increased bacterial resistance to phage infection, whilst ameliorating the costs associated with resistance mutations. As a result, the stronger selection for increased phage infectivity destabilises the coevolutionary interaction between bacteria and phage, and may therefore result in phage extinction in enriched environments [12]. However, we found the opposite; the likelihood of phage extinction was increased at low resource levels.

Whilst these studies are not directly comparable (our experiment was conducted different culture media and at different population sizes) these broadly contrasting outcomes are likely to be due to differences in the magnitude of the resource manipulations in each study. In our study we implemented a much more minimal low-resource condition (100-fold dilution of KB growth media compared with a maximum 10-fold dilution of LB media used by Zhang and Buckling [12]). Under comparable (i.e. static growth) conditions we observed far higher rates of extinction in low resource conditions (3 out of 6 replicates) than the more intermediate resource levels used in the previous study (0.1 and 0.3LB), where no extinctions were observed. Extinction rates under high resources were more similar between experiments (1 out of 6 replicates in our static 1 KB treatment compared to 2 out of 8 replicates in their 2.7LB treatment). High rates of phage extinction may therefore be a result of the ecological effects of very low resource environments, suggesting that the stability of coevolutionary interactions may be maximised under intermediate resource levels. Interestingly, however, the mechanisms driving extinction at high and low resources are likely to derive from the same fundamental process; limiting the potential of phage to evolve infectivity in response to bacterial resistance. Under high resource environments accelerated rates of antagonistic coevolution increases demand for novel resistance and infectivity mutations. In contrast, low resource levels sustained smaller population sizes which can limit the supply of resistance and infectivity mutations [23]. Because phage infectivity is known to require several mutational steps to overcome a single bacterial resistance mutation [11, 24] both increasing demand or limiting supply of mutations is likely to disproportionately constrain the ability of phage to adapt compared with bacteria and thus lead to an imbalance in bacteria-phage coevolution and ultimately phage extinction.

Under very low resources these effects are likely to be further exacerbated by the impact of reduced host diversity due to more stringent bottlenecks, which can result in coevolution-induced phage extinction through stochastic loss of susceptible bacteria [25]. An important caveat of our experimental design is that the magnitude of bottlenecking imposed by experimental transfers varies dependent on the population density supported by each treatment, such that the reduced population sizes in low resource conditions also translate to a more stringent bottleneck. Furthermore, low resource conditions have been shown to inhibit the establishment of bacteria-phage interactions, reducing connectivity of the population (i.e. lower density of realised interactions) and thus further increasing vulnerability to extinction [26]. This mechanism is supported by our resistance assays which show that phage extinction in low resource treatments was associated with high levels of bacterial population-level resistance (Fig. 3a). Moreover, populations in which phage were maintained experienced reinvasion of highly susceptible bacteria, displacing resistant host genotypes (Fig. 3b, d).

As predicted, population mixing was shown to increase the risk of phage extinction. Higher encounter rates in mixed environments, which stimulate more intense coevolution [19], increased the risk of phage extinction consistently across high and low resource supply levels. Population mixing may act to intensify the coevolutionary imbalances promoted in each resource condition, which limit phage adaptation, thereby increasing the likelihood of phage extinction. Conversely, static conditions provide spatial refuges for susceptible bacteria by reducing dispersal and lowering encounter rates [27], thereby limiting selection for bacterial resistance. In turn, this alleviates selection for increased phage infectivity and as a result may stabilise the coevolutionary interaction, promoting co-existence. Bacterial spatial refuges may be ‘self-organised’ in nature: Modelling of bacteria-phage populations demonstrates that density-dependent mechanisms, such as biofilm formation, may promote formation of phage-exclusion zones [28]. High phage densities could develop on the boundaries, but do not penetrate such zones, enabling susceptible bacterial genotypes to persist thereby promoting stable bacteria-phage coexistence.

## Conclusions

Our results suggest that coevolution-induced phage extinction is promoted by abiotic conditions which destabilise the symmetry of antagonistic coevolution. This highlights the importance of feedbacks between ecological processes and the co-evolutionary interactions embedded within communities. We currently understand little about the effects of environmental changes across space and time on the stability of microbial populations, or the impact of short term responses to such changes on the long-term evolutionary trajectory of these communities. Whilst a predator-prey style paradox of enrichment may apply to bacteria-phage interactions in some situations, it is clear that the multivariate ecological and evolutionary effects of resource enrichment or depletion depend on the broader ecological and evolutionary context.
